# The Acidic Stress Response of the Intracellular Pathogen *Brucella melitensis*: New Insights from a Comparative, Genome-Wide Transcriptome Analysis

**DOI:** 10.3390/genes11091016

**Published:** 2020-08-28

**Authors:** David Kornspan, Tamar Zahavi, Mali Salmon-Divon

**Affiliations:** 1 Department of Bacteriology, Kimron Veterinary Institute, Bet Dagan 50250, Israel; 2 Genomic Bioinformatics Laboratory, Department of Molecular Biology, Ariel University, Ariel 40700, Israel; tamar.harel1@mail.huji.ac.il (T.Z.); malisa@ariel.ac.il (M.S.-D.); 3 Adelson School of Medicine, Ariel University, Ariel 40700, Israel

**Keywords:** *Brucella melitensis* 16M, acidic stress, transcriptomic analyses, RNA-seq, virulence

## Abstract

The intracellular pathogenic bacteria belonging to the genus *Brucella* must cope with acidic stress as they penetrate the host via the gastrointestinal route, and again during the initial stages of intracellular infection. A transcription-level regulation has been proposed to explain this but the specific molecular mechanisms are yet to be determined. We recently reported a comparative transcriptomic analysis of the attenuated vaccine *Brucella melitensis* strain Rev.1 against the virulent strain 16M in cultures grown under either neutral or acidic conditions. Here, we re-analyze the RNA-seq data of 16M from our previous study and compare it to published transcriptomic data of this strain from both an in cellulo and an in vivo model. We identify 588 genes that are exclusively differentially expressed in 16M grown under acidic versus neutral pH conditions, including 286 upregulated genes and 302 downregulated genes that are not differentially expressed in either the in cellulo or the in vivo model. Of these, we highlight 13 key genes that are known to be associated with a bacterial response to acidic stress and, in our study, were highly upregulated under acidic conditions. These genes provide new molecular insights into the mechanisms underlying the acid-resistance of *Brucella* within its host.

## 1. Introduction

Many microorganisms can survive and grow under stressful conditions and extreme ecological niches, including in highly acidic microenvironments [1,2,3]. Since acidic stress has significant ramifications in agriculture, the food industry, and human health, the molecular mechanisms underlying acid tolerance in pathogenic bacteria have been rigorously investigated [1,2,3,4,5]. Such mechanisms are highly diverse and may include, for instance, various changes in cell structure, metabolism, and transport patterns [1,2,3]; proton pumps that maintain a tolerable internal pH [1,2,3,6,7]; the induction of specialized repair mechanisms [1,8]; the generation of ammonia through glutaminase and arginine deiminase pathways [6,9,10,11]; the activation of urease [5,12]; and changes in the lipid composition of the membrane [13]. Studies of the molecular mechanisms that enable bacteria to cope with acidic stress have distinguished between two acid-related response systems: the acid resistance (AR) system response to extreme acidic stress (pH 1–3, characterizing, for example, the pH in the human stomach), and the acid tolerance response (ATR) system to mild or moderate acidic stress (pH 4–5, characterizing, for example, the intracellular vacuole of the host); whereas the former involves mechanisms that prevent the intracellular pH from falling to life-threatening levels (e.g., amino acid-dependent systems, urease, or repair mechanisms), the latter involves mechanisms that maintain intracellular pH homoeostasis, such as F_1_Fo-ATPase activity [1].

Understanding the molecular mechanisms responsible for acid tolerance may be especially important in the case of pathogenic bacteria. One such species is the facultative intracellular bacteria *Brucella melitensis*, which is responsible for brucellosis [14,15]: a zoonotic disease that causes abortions and sterility in animals and a severely debilitating febrile illness in humans [14,15]. The most common route of entry of *Brucella* into the human body is the gastrointestinal route, usually as a result of consuming contaminated raw milk or its products [16,17]. To colonize within the host, the bacteria must tolerate the strong acidic environment of the stomach (pH 1.5–3)—a process in which urease has been suggested to play a significant role [17]. However, once they pass this barrier, the bacteria need to survive within various types of host cells [18,19] so as to be inaccessible to the humoral immune response of the host [20]. To this end, following their uptake by the host cells, *Brucella* create the *Brucella*-containing vacuole (BCV)—an intermediate acidic intracellular niche in which they reside and multiply [21,22]. The acidification of the BCV is essential for inducing the type-IV secretion system (T4SS; [23,24]) that is encoded by the VirB locus and plays a crucial role in the intracellular survival and replication of *Brucella* within the host cells [25,26]. This acidification is, therefore, crucial for the pathogenesis of *Brucella* [24] and requires the bacteria to cope with a rapid and significant drop in their microenvironmental pH; indeed, the pH in phagosomes containing live *B. suis* has been shown to decrease to 4.0 within 1 h post-infection (p.i.), and this pH is maintained for at least 5 h [24].

Recent comparative transcriptome analyses revealed complex, transcription-level regulation networks of *Brucella* within the acidic environment of the BCV. For instance, Liu et al. [27] highlighted the critical role of the response regulator OtpR in regulating the metabolism and virulence of *B. melitensis* under acidic stress, and, in a different work, Liu et al. [28] revealed the role of the gene BMEI1329, which encodes a two-component response regulator, in the acid-resistance and virulence of *B. melitensis*. In a recent comparative transcriptomic study [29], we reported differences between two *B. melitensis* strains—the virulence-attenuated vaccine strain Rev.1 and the more virulent strain 16M—in cultures grown under either neutral or acidic conditions. Similar to the abovementioned transcriptomic studies, our study highlighted the involvement of specific genes and genetic systems in the pathogenicity of *B. melitensis* under acidic conditions. However, a comprehensive, in-depth investigation of the mechanisms that enable *B. melitensis* to cope specifically with acidic environments is still lacking, and how the bacteria trigger their environmental adaptations to acidic conditions is yet to be determined.

In the present work, we investigate the acid tolerance (pH 4–4.5) of the virulent *B. melitensis* strain 16M, focusing on genetic expression and using a combination of RNA-seq and comprehensive statistical analyses. We integrate this analysis with the published transcriptomic analyses of *B. melitensis* 16M in an in cellulo HeLa cell culture model [30] and in an in vivo goat supramammary lymph node model [31]. In this way, we aim to elucidate the mechanisms that potentially allow this pathogen to cope with the rapid decrease in pH within its host.

## 2. Materials and Methods

### 2.1. Public Data Sets

To identify genes associated with the response to acid stress, we re-analyzed our previously reported data set PRJNA498082 [29], which can be downloaded from the Sequence Read Archive (SRA; [32]) database. This data set contains the comparative transcriptome analysis of the *B. melitensis* Rev.1 vaccine strain against the virulent reference strain 16M in cultures grown for 4 h under either neutral (pH 7.3) or acidic (pH 4.4) conditions. To identify the specific genes that respond to acidic stress in *B. melitensis* 16M, we focused on the RNA-seq data of this strain only.

### 2.2. Data Analysis

To compare our data with those from the in cellulo HeLa cell study by Rossetti et al., [30] we downloaded the lists of genes that responded to acidic stress from the online supplementary data of that study. To compare our data with those from the in vivo infection model by Boggiatto et al., [31] we used the raw fastq files from that study, which were kindly provided by Steven C. Olsen and Darrell O. Bayles (U.S. Department of Agriculture, Agricultural Research Service, National Animal Disease Center, Infectious Bacterial Diseases Research Unit). These files contain data from five cultured 16M replicates, four replicates of bacteria recovered from the supramammary lymph nodes of experimentally infected goats 4 w p.i., and three replicates isolated from the experimentally infected goats 38 w p.i. The initial RNA-seq analysis, including adapter trimming, read mapping to reference genome, and gene counts, was conducted as previously described [29]. For the combined differential expression analysis of our previously reported 16M data set and of the in vivo infection model, we employed the edgeR [33] and limma [34] R packages. First, genes, whose total counts were fewer than 1 cpm in at least three samples, were filtered out and the remaining gene counts were normalized using the trimmed mean of M values (TMM) method [35] followed by a Voom transformation [36]. A multidimensional scaling (MDS) analysis was used to visualize the level of similarity between samples and to detect possible outliers.

### 2.3. Statistical Analysis

To detect differentially expressed genes, a linear model was fitted using weighted least squares for each gene, and the comparison of interest was extracted from the fit. To correct for batch effect, we included a batch term in the model matrix. Genes with false discovery rate (FDR) < 0.05 and fold change > 2 were considered as differentially expressed (DE). The moderated F statistic was used to extract genes that are DE in at least one of the three analyzed groups (avoiding the error inherent in performing multiple tests). To sort genes based on their abundancy, we computed the reads per kilobase per million (RPKM) values to normalize for both sequencing depth and gene length. Unless indicated otherwise, the supervised hierarchal clustering of DE genes was accomplished by using “Euclidean” as the distance measure and “complete” as the linkage method. Genes of each cluster were subjected to a gene ontology enrichment analysis using ShinyGO v 0.61 [37]. The genes were categorized to clusters of orthologous group categories (COGs) by using the information stored on the EggNOG database [38]. The protein–protein interaction network was detected and visualized by using the STRING tool [39].

### 2.4. Reverse-Transcriptase PCR (RT-PCR)

The total RNA was isolated using the RNeasy Mini Kit (Qiagen, Hilden, Germany) with a DNase treatment (Qiagen). RNA was eluted from the column using RNase-free water. RNA quality was measured using Bioanalyzer (Agilent, Waldbronn, Germany). To confirm the RNA-seq results, five upregulated or downregulated *B. melitensis* 16M genes from our RNA-seq analysis [29] were selected, and an RT-PCR was used to confirm the expression changes of these genes under both acidic and neutral pH (see [29] for details). PCR primers were designed by using Primer3web version 4.1.0 [40] and are listed in Supplementary Table S1. Complementary DNA (cDNA) was obtained by the reverse transcription of 850 ng total RNA using a GoScript™ Reverse Transcription System kit (Promega, Madison, WI, USA), according to the protocol recommended by the manufacturer. PCR reactions were conducted using the Applied Biosystems 7500 Fast platform in a final reaction volume of 20 µL containing 20 ng of cDNA template, 10 µL of Fast SYBR^®^ Green Master Mix (Applied Biosystems™, Thermo Fisher Scientific, Vilnius, Lithuania), and 1 µL of primer mix. The PCR result was considered positive if there was an amplification within 30 cycles. All reactions were run in triplicate and the reference gene 16S rRNA was amplified in a parallel reaction for normalization.

## 3. Results

We re-analyzed our previously published RNA-seq data [29] to conduct a comprehensive comparative transcriptomic analysis of the gene expression profiles of *B. melitensis* 16M, grown under either acidic conditions (pH = 4.4; “acidic” group) or neutral pH conditions (pH = 7.3; “neutral pH” group). Below, we first characterize the gene expression profile of each experimental group and then list the genes that were DE between these groups. Next, we compare between the reported gene expression of *B. melitensis* 16M in the in cellulo HeLa cell culture model [30] and in the in vivo goat supramammary lymph node model [31]. Finally, we report the potential genes associated with the specific responses of *B. melitensis* 16M to acidic stress.

### 3.1. Gene Expression in B. melitensis 16M Grown under Either Acidic or Neutral pH Conditions

**Figure 1:**
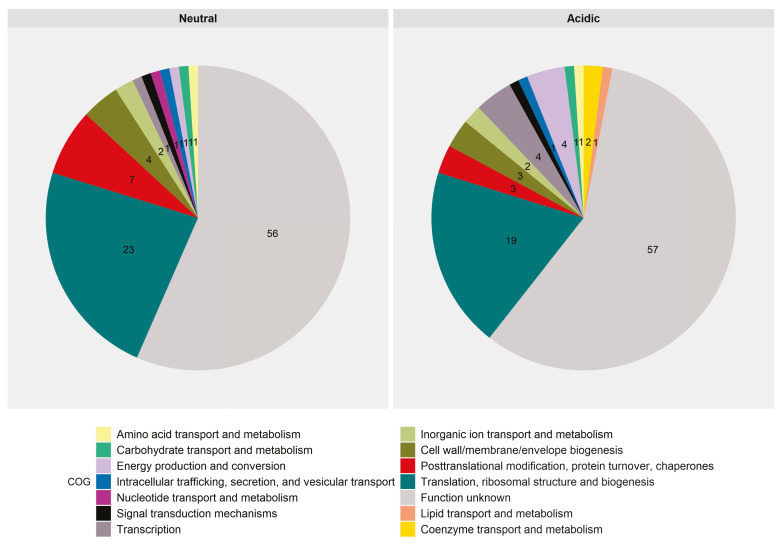
Genes transcribed in *Brucella melitensis* 16M grown under either neutral (**A**) or acidic (**B**) pH conditions. For each sample set, the 100 genes with the highest levels of expression—based on counts normalized to gene size and library size (RPKM)—were categorized by their Clusters of Orthologous Groups (COG) category to generate the percentages shown in the pie charts. COG categories were retrieved from the EggNOG database [38].

In total, we analyzed the expression of 3356 genes in the 16M strain. The top 100 genes, ranked by the most abundant expression from each experimental group (based on counts normalized to gene and library size) were categorized by functional annotation based on COGs. These top-expressed genes probably reflect the most dominant expressed genes of *B. melitensis* 16M under each environmental pH (acidic or neutral) and are, therefore, of particular interest. The lists of annotated genes are included in Supplementary Tables S2 and S3. For the top 100 expressed genes of known functions in the neutral pH group, the following categories were most represented: translation, ribosomal structure, and biogenesis (23%); posttranslational modification, protein turnover, chaperones (7%); and cell wall/membrane/envelope biogenesis (4%) (Figure 1A). For the top 100 expressed genes of known functions in the acidic group, the following categories were most represented: translation, ribosomal structure, and biogenesis (19%); transcription (4%); and energy production and conversion (4%) (Figure 1B).

**Figure 2:**
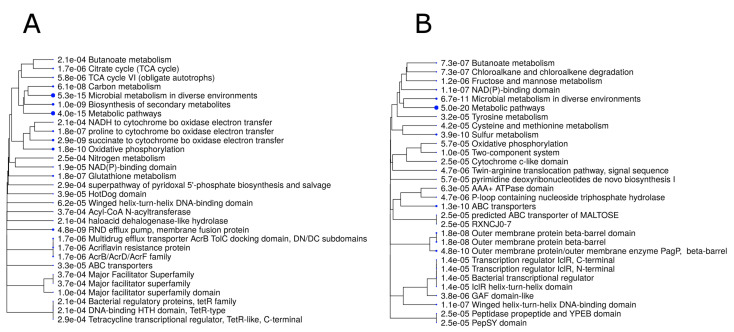
A hierarchical clustering tree, summarizing the correlations between significant pathways among the list of upregulated (**A**) and downregulated (**B**) genes. Pathways with many shared genes are clustered together. Larger dots indicate more significant *p*-values.

In total, 760 genes in the *B. melitensis* 16M strain were DE (FDR < 0.05, fold change ≥ 2) between bacteria grown under neutral and acidic pH conditions, including 360 upregulated and 400 downregulated genes in the acidic group, as compared with the neutral pH group (Supplementary Table S4). These 760 genes were then subjected to a gene ontology (GO) enrichment analysis, which revealed that the most significant biological pathways among the upregulated genes were microbial metabolism in diverse environments, metabolic pathways, oxidative phosphorylation, and Resistance-Nodulation-Division (RND) efflux pump, whereas the most significant biological pathways among the downregulated genes were metabolic pathways, sulfur metabolism, and ABC transporters (Figure 2).

**Figure 3:**
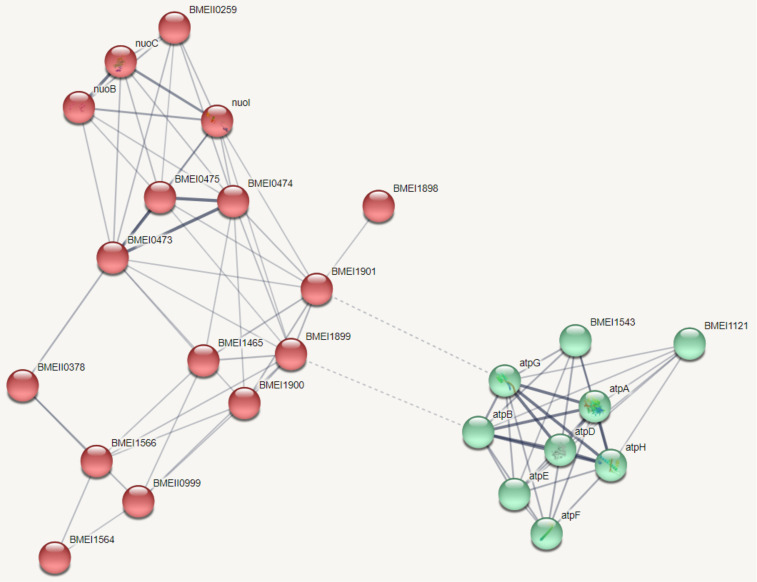
Protein–protein interaction network of 25 proteins whose gene expression was different between the neutral and acidic pH groups. In this network, 22 proteins are related to oxidative phosphorylation processes. The proteins in the network were divided into two clusters based on the distance matrix obtained from their global confidence scores. The proteins in the right cluster (green) are mostly ATP synthase subunits while the proteins in the left cluster (red) are mainly electron carriers. Inter-cluster edges are represented by dashed-lines.

Next, we explored the protein–protein interaction networks within the 760 DE genes, using only experimentally validated associations. We found a network of 25 proteins, of which 22 were related to oxidative phosphorylation processes (Figure 3).

### 3.2. The Effects of Acid Stress on Gene Expression in B. melitensis 16M, as Compared with Its Previously Reported Transcriptional Profile in an In Cellulo Hela Cell Model

**Figure 4:**
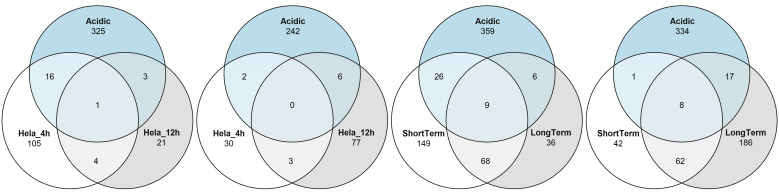
Comparative analysis between expressional profiles of 16M grown in a “pure” acidic environment versus the intracellular niche of its host. (**A**) Genes found to be downregulated in an in cellulo HeLa cell model. (**B**) Genes found to be upregulated in an in cellulo HeLa cell model. (**C**) Genes found to be downregulated in an in vivo goat supramammary lymph node model. (**D**) Genes found to be upregulated in an in vivo goat supramammary lymph node model.

To adapt to the environmental changes and cope with the cellular defense mechanisms within the cells of their hosts, intracellular pathogens execute a coordinated regulation of the expression of specific genes [41]. Rossetti et al. [30] characterized the transcriptional profile of *B. melitensis* 16M at two time points following infection of HeLa cells, namely, at 4 h p.i. and at 12 h p.i. (referred to as the adaptation and the replicative periods, respectively). The authors identified 161 and 115 DE genes at 4 h and 12 h p.i., respectively, as compared with bacteria grown in vitro. Next, the authors showed that (a) most of these DE genes are involved in growth- and metabolism-related processes of the pathogen, and (b) most of these genes were downregulated at 4 h p.i. and upregulated at 12 h p.i. It is reasonable to assume that the bacteria encounter and need to cope with the various environmental stresses within their host cells—including acidic stress—soon after infecting the host, namely, at the earlier time point. Hence, to investigate the differences between the gene expressional profile of 16M grown in a “pure” acidic environment versus the intracellular niche of its host, we compared the list of DE genes published by Rossetti et al. to the list of DE genes in our study (acidic group). This comparison revealed that only a minority of DE genes are common to both lists (Figure 4A,B, Supplementary Table S5), and that these genes are related to the following COG categories: Inorganic ion, nucleotide transport and metabolism; transcription; and energy production and conversion.

### 3.3. The Effects of Acid Stress on Gene Expression in B. melitensis 16M, as Compared with Its Previously Reported Transcriptional Profile in an In Vivo Infection Model

Boggiatto et al. [31] examined the transcriptional profile of *B. melitensis* 16M RNA obtained from the supramammary lymph node of experimentally infected goats at 4 w p.i. and at 38 w p.i. (referred to as short- and long-term infection, respectively). To investigate the differences between the gene expressional profile of 16M grown in a “pure” acidic environment versus the natural in vivo environment of the host, we compared the lists of DE genes in the in vivo infection study (considering both short- and long-term infection) of Boggiatto et al. to the list of DE genes in our study (acidic group). After adjusting for batch effect, we visualized the level of similarity of different samples from the two experiments by using an MDS plot, which revealed four separate groups, emphasizing the reproducibility of the analyzed data (Figure S1).

In total, only 17 DE genes were common to all three conditions (acidic, short-term, and long-term), while 359 and 334 downregulated and upregulated genes, respectively, were unique to the acidic group (FC = 2, FDR = 0.05, moderated *t* tests; Figure 4C,D, Table S6). These genes belong to microbial metabolism in diverse environments, metabolic pathways, oxidative phosphorylation, ABC transporters and carbon metabolism.

**Figure 5:**
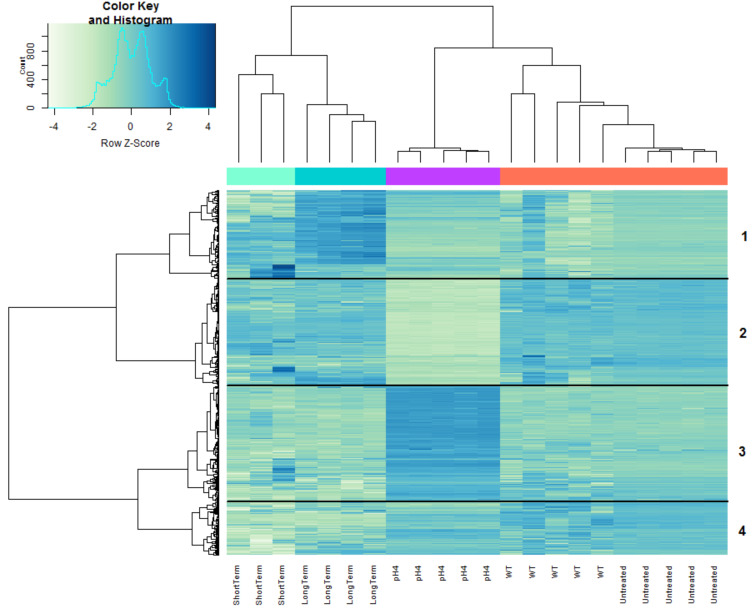
Heatmap showing the expression profiles of 1256 genes that were differentially expressed (DE) (green: downregulated; blue: upregulated) in at least one of the analyzed groups (neutral pH: red bar; acidic pH: purple bar; short-term infection: turquois bar; and long-term infection: green bar). The neutral pH group was subdivided to either “WT”—control samples provided by Boggiatto et al. [29] or “Untreated”—control samples from our study [27]. Rows indicate genes and columns indicate bacterial samples. Green and blue pixels indicate downregulated and upregulated genes, respectively. The hierarchical clustering was generated by using the 1-Pearson correlation as the distance measure and “ward.D2” as the linkage method. Genes were categorized into four clusters based on the generated dendrogram.

A heatmap of 1256 genes that were significantly DE in at least one of the analyzed groups (moderated F test, FC = 2, FDR = 0.05), categorized into four clusters, is presented in Figure 5. Clusters 1 and 4 represent the differences in gene expression between in vivo and in vitro conditions, while Clusters 2 and 3 include genes that were significantly down- and upregulated, respectively, in the acidic group and showed the opposite trend in the in vivo model. These clusters clearly demonstrate the unique expressional profile of *B. melitensis* 16M under acidic conditions, as compared with the expressional profile of this pathogen within its host. A GO analysis of the genes within these two clusters revealed the enrichment of the following pathways: in Cluster 2—metabolic pathways, ABC transporters, and sulfur metabolism; and in Cluster 3—metabolic pathways, oxidative phosphorylation, biosynthesis of secondary metabolites, succinate to cytochrome bo oxidase electron transfer, carbon metabolism, and RND efflux pump.

### 3.4. Key Genes in B. melitensis 16M That Are DE Specifically in Response to Acidic Stress

To elucidate the genes in *B. melitensis* 16M that are specifically up- or downregulated in response to acidic stress, we analyzed the genes in 16M that were DE between the acidic and neutral pH groups in our previous study, but that were not DE in the in cellulo or in vivo models described above. This approach revealed 588 genes that are potentially induced or repressed specifically by acidic stress (Supplementary Table S7), and that may shed light on the mechanisms by which *B. melitensis* 16M copes with such stress. Categorizing these 588 genes by their functional annotation (based on COGs) revealed that the most represented categories were energy production and conversion (7%), cell wall/membrane/envelope biogenesis (6%), and inorganic ion transport and metabolism (5%) (Figure S2). Annotating these genes revealed that 48 genes are involved in the mitigation of oxidative stress, transport, ATP synthesis, cell cycle, cytochrome oxidase activity, and virulence; of these 48 genes, 38 were upregulated and 10 were downregulated by acidic stress (Supplementary Table S8). Among the upregulated genes, 13 genes were highly upregulated key genes that are associated with bacterial response to acidic stress (Table 1), and we assume that they play a vital role in the response of *B. melitensis* 16M to acidic stress.

### 3.5. RT-qPCR Validation of the RNA-Seq Results

To ensure technical reproducibility and to validate the data generated from the RNA-seq experiment, we conducted a real-time qPCR analysis of five selected highly DE genes (Supplementary Table S1) from *B. melitensis* 16M, grown under either acidic or neutral pH conditions. The mRNA levels of all genes obtained by the RT-qPCR were in high accordance with those obtained by our RNA-seq analysis (Table 2).

## 4. Discussion

To elucidate the molecular mechanisms underlying the tolerance of the *B. melitensis* 16M virulent strain to acidic stress, we re-analyzed our previous RNA-seq data of 16M, grown under either acidic or neutral pH conditions [29]. The acidic conditions reflect the environmental pH during infection via the gastrointestinal infection route [17] and during the initial stages of intracellular infection, namely, the initial environmental pH in the BCV [21,24,42]. The survival of the pathogen during these stages is crucial for the establishment of infection and for further replication within the host [26,43]. Therefore, identifying the key genes that are potentially involved in the adaptation and survival of *B. melitensis* 16M under acidic stress is of high importance.

Among the 588 unique genes that were DE between the acidic and neutral pH groups were five genes encoding ATP synthase subunits, which were significantly downregulated in our study under acidic conditions. The F_1_F_0_-ATPase machinery located on the plasma membrane can operate as either ATP synthase or ATPase [1,44], and it is reasonable to assume that the acid-induced downregulation of genes that are involved in ATP synthesis decreases the exergonic entry of protons into the bacterial cell, thus providing some degree of resistance against acidic stress.

Among the 13 genes that are known to be involved in bacterial responses to acidic stress, and which were significantly upregulated in our study under acidic conditions, were six genes that belong to the urease operon. Urease is a nickel-dependent metalloenzyme that catalyzes the hydrolysis of urea into ammonia (NH_3_) and carbon dioxide (CO_2_) [45]. In several bacterial species (e.g., *Helicobacter pylori*, *Streptococcus salivarius*, and *Staphylococcus aureus*), urease plays an important role in the bacterial acid-response network by generating ammonia, which protonates into ammonium (NH_4_^+^), thus consuming intracellular protons and increasing the intracellular pH [46,47,48]. Most members of the genus *Brucella* show strong urease activity [17,49]. In *Brucella abortus*, specifically, urease-producing strains are resistant to strong acid conditions in vitro, whereas urease-negative mutants are susceptible to acid treatment and were killed more efficiently during transit through the stomach in an in vivo mouse model [17], thereby, it had been suggested that urease protects *Brucella* during their passage through the stomach [17]. The significant upregulation of six genes of the urease operon in *B. melitensis* 16M under moderate acidic stress conditions may suggest that *Brucella* uses urease activity not only during extreme acidic stress, but also in the crucial initial stage of infection, within the intracellular niche of the BCV. Notably, the urease activity as a function of pH was already demonstrated in intact *H. pylori* two decades ago and revealed a rapid 10-fold increase in urease activity when the pH dropped below 6.5. In *H. pylori*, urease activity remained relatively constant between pH 6.5 and pH 2.5 [48]. In *Brucella*, our results indicate that the urease operon is induced at pH 4; its activity at this pH remains to be confirmed by further biological experiments. In a similar vein, our analysis revealed that the gene BME_RS15250, which encodes nucleoside deaminase, is highly upregulated in *B. melitensis* 16M under moderate acidic stress. Both deiminase and deaminase systems can produce ammonia [1,9], suggesting an acid-resistance mechanism similar to that induced by urease. Indeed, Sun et al. [49] showed that although adenosine deamination increased the survival of *Escherichia coli* under extreme acidic conditions, the expression of the *add* gene encoding adenosine deaminase was also increased at pH 5.5 [50], indicating the important additional role of this enzyme in the adaptation to moderate pH.

Three other genes that were upregulated in 16M under acidic stress encode four major facilitator superfamily (MFS) efflux pumps. These pumps are membrane protein complexes that are conserved in all living organisms [51,52]. In several pathogenic bacteria (e.g., *Salmonella typhimurium*, *Listeria monocytogenes*, and *Vibrio cholera*), efflux pumps were shown to be involved in antibiotic extrusion and contribute to host colonization, intracellular survival, resistance to stress, and biofilm formation [53,54,55]. In *S. typhimurium*, efflux pumps play an important role in the invasion process and survival within macrophages and intestinal epithelial cells [56]. The *emrKY* genes of *Shigella flexneri*, which encode the MFS efflux pump EmrKY, have recently been shown to be specifically and highly induced in *Shigella*-infected macrophages and are activated in response to a combination of high K+ and low pH [52]. Notably, Xu et al. [57] demonstrated the role of an MFS transporter from the fungus *Penicillium funiculosum* in the adaptation capacity to extreme acidic stress and in intracellular pH homeostasis. It is possible that, similar to *S. flexneri* and *P. funiculosum*, *B. melitensis* 16M upregulates these genes as a secondary mechanism for coping with acidic stress.

Another gene that was upregulated in *B. melitensis* 16M under acidic conditions is BMEI0564, which encodes the molecular chaperone DjlA—a member of the DnaJ/Hsp40 family. Molecular chaperones facilitate protein folding and prevent protein denaturation, and they are involved in various cellular processes, such as DNA replication, RNA transcription, and bacterial growth [58]. Under acidic conditions, partially unfolded proteins may emerge and molecular chaperones may stabilize them to prevent their acid-induced aggregation [59]. For instance, DjlA from *Legionella dumoffii* was shown to play an important role in intracellular growth, organelle trafficking, and resistance to acidic, oxidative, osmotic, and heat stresses [60]. Thus, the upregulation of BMEI0564 under acidic stress may play an important role in preventing the irreversible aggregation of misfolded proteins, thus contributing to bacterial survival in the host. Notably, the BME_RS14625 gene, encoding the acid stress chaperone HdeA, was downregulated in our study. This finding may be explained by previous studies in *E. coli*, which demonstrated that this chaperone is only activated during extreme acidic stress (pH < 3; [61,62]).

Two other genes that were significantly upregulated in *B. melitensis* 16M under acidic stress were BMEII0294 and BMEI1248, which encode two glutathione S-transferases (GSTs). GSTs are evolutionarily conserved enzymes that are important in the detoxification of various xenobiotic compounds [63,64] and protect cells from oxidative stress by detoxifying some of the secondary reactive oxygen species (ROS), including superoxide anions, hydroxyl radicals, and hydrogen peroxide [63,64]. In *Proteus mirabilis*, a glutathione S-transferase B1-1 null mutant was found to be more sensitive to oxidative stress (in the form of H_2_O_2_) than its wild-type counterpart. Accordingly, it was suggested that this GST plays an active role in protecting against oxidative stress [65]. In *E. coli*, pH regulates the expression of genes encoding for proteins that are involved in oxidative stress; therefore, acidic stress and oxidative stress have been assumed to be strongly correlated [66], such that acid conditions accelerate the production of oxygen radicals, thus inducing a partial oxidative stress response [66]. It is possible that the potential correlation between acidic and oxidative stress leads to the up-regulation of GSTs in *B. melitensis* 16M under acidic stress.

Three other genes that were significantly upregulated in *B. melitensis* 16M under acidic stress were BMEII0604, BMEII0606, and BMEII0883, which encode iron transporters. Connections between acid stress and metal ion homeostasis have been previously reported in Group B streptococcus (GBS; [67]). Santi et al. [68] reported higher expression levels of several Mn^2+^ and Fe^2+^ transporters of GBS upon shifting from pH 7 to pH 5.5. The upregulation of such transporters was attributed to an increased need for essential metals during acidic stress [67]. The upregulation of iron transporters in *B. melitensis* under moderate acidic conditions strengthens this assumption. 

As compared with 16M grown under neutral pH, the acidic pH group showed a specific downregulation of five key genes (BME_RS13825, BMEI0168, BMEII0925, BMEI0072, BMEI0313) that encode for two DNA translocase proteins and three cell-division proteins, which participate in critical stages of the bacterial cell cycle [69,70]. This finding may indicate that, under acidic stress, 16M temporarily arrests cell division and activates pathways to bypass or repair damaged DNA—a mechanism known to take place in many bacterial species during the adaptive program known as the “SOS response” [41,71]. Support for this conclusion comes in the form of the significant upregulation of BMEI1247, BMEI0068, and BME_RS11465 (Supplementary Table S7), which encode ribonuclease T, exodeoxyribonuclease III, and GIY-YIG nuclease family protein, respectively, and play an important role in DNA-repair processes [72,73].

Finally, the DE genes BMEII0025, BMEII0027, and BMEII0028, which encode the T4SS proteins VirB1, VirB3, and VirB4, respectively, were significantly upregulated in 16M under acidic stress, as compared with neutral pH conditions. The *Brucella* VirB T4SS, which consists of 12 genes (VirB1–12) and whose induction requires the acidification of the BCV [24,26], is a key virulence factor that plays an important role in mediating intracellular survival and in manipulating the host immune response to infection [25,26,74]. In macrophages, the acidified environment has been shown to induce the expression of the VirB operon, which interacts with the endoplasmic reticulum to neutralize the pH of the phagosome [21,75], thus enabling the replication and establishment of *Brucella* within its host.

Notably, some of the highly important ATR and AR systems discussed above with reference to pathogenic bacteria are also similar to the response of health-associated bacteria to acidic stress. For instance, lactic acid bacterial species use urease activity to counteract acidic stress [76]. Alteration in membrane fluidity, fatty acid distribution, and cell integrity were shown to be common mechanisms utilized by the probiotic bacteria *Lactobacillus casei* to withstand severe acidification and to reduce the deleterious effect of lactic acid on the cell membrane [77]. Bifidobacteria, which are an important part of normal intestinal microbiota of various mammalian species and are the best characterized and widely commercialized probiotics [78], were shown to discharge H^+^ by H^+^-ATPase, block H^+^ by the cell membrane and cell wall, neutralize H^+^ by alkalinity products, and communicate intercellularly via quorum sensing, in order to cope with acidic stress [79].

## 5. Conclusions

Through a comprehensive comparative transcriptomic analysis of *B. melitensis* 16M, grown under either acidic or neutral pH conditions, together with the published data of the expressional gene pattern of this bacterium in in cellulo and in vivo models, we found several genes that play key roles in various crucial pathways in *Brucella* that are either up- or downregulated under acidic stress. We suggest that these genes—and, especially, those listed in Table 1—are involved specifically in the molecular mechanisms underlying the *B. melitensis* 16M response to acidic environments. Further characterization, through mutation and knockout experiments, is required to conclusively determine the role of these genes in acid resistance.

## Figures and Tables

**Table 1:** Key genes potentially involved in the response of *B. melitensis* 16M to acidic stress (false discovery rate (FDR) < 0.001 for all listed genes).

Gene ID	Gene	Fold Change
BMEI1655	urease accessory protein ureD 1	14.38
BME_RS08240	urease subunit gamma	11.29
BMEI1653	urease subunit beta	11.22
BMEI1652	urease subunit alpha 1	7.86
BMEI1650	urease accessory protein UreF 2	5.37
BMEI0642	urea transporter	4.49
BMEI0556	MFS transporter	4.33
BMEII0027	type IV secretion system protein VirB3	2.92
BMEII0025	type IV secretion system protein VirB1	2.70
BMEI0181	MFS transporter	2.57
BMEII0280	MFS transporter	2.53
BMEII0028	type IV secretion system protein VirB4	2.2
BMEI0564	molecular chaperone DjlA	2.07

**Table 2:** Validation of selected genes by RT-qPCR.

Gene ID	Gene	Fold Change (Acidic vs. Neutral pH)	*p*-Value (*t*-Test)
BMEI1900	cytochrome o ubiquinol oxidase subunit I	4.93	<0.001
BMEII1119	MFS transporter	12.7	0.02
BMEII0025	type IV secretion system protein VirB1	81.8	0.01
BME_RS13825	DNA translocase FtsK	−17.7	<0.001
BMEI2002	molecular chaperone DnaK	−12	0.037

## Supplementary Materials

The following are available online at https://www.mdpi.com/2073-4425/11/9/1016/s1, Table S1: RT-PCR primers used in this study for validation, Table S2: The top 100 genes ranked by the most abundant expression from 16M grown under neutral conditions, Table S3: The top 100 genes ranked by the most abundant expression from 16M grown under acidic conditions, Table S4: DE genes (FDR < 0.05, fold change ≥ 2) between 16M grown under neutral and low-pH conditions, Table S5: The list of the unique and common genes, ours and Rossetti et al. DE genes, Table S6: Genes differentially expression in low pH, in vivo short and long infection, as compared with untreated bacteria, Table S7: The unique acidic up- and down-regulated genes of *B. melitensis* 16M, Table S8: Key genes in *B. melitensis* 16M that are DE specifically in response to acidic stress, Figure S1: Similarities between bacterial samples visualized using an MDS analysis, Figure S2: COG categories of genes (n = 588) in *B. melitensis* 16M that are specifically up- or downregulated in response to acidic stress.
