# Supplementary material for: The Acidic Stress Response of the Intracellular Pathogen Brucella melitensis: New Insights from a Comparative, Genome-Wide Transcriptome Analysis
Source: Genes (Basel). 2020 Aug 28;11(9):1016. doi: 10.3390/genes11091016 (PMC7563570; doi:10.3390/genes11091016)
Supplement: Supplementary file 1 [file genes-11-01016-s001.zip › Supplemetary figures revision new.docx]

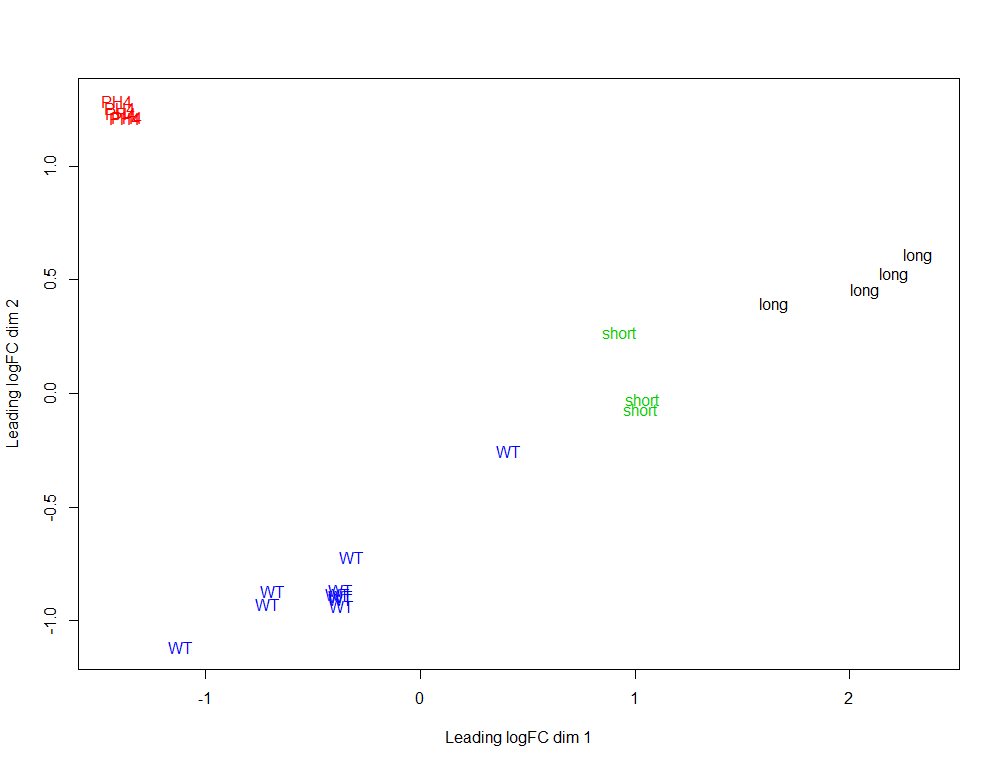


Figure S1: Similarities between bacterial samples visualized using an MDS analysis. Relative distances between the samples were projected onto a 2-dimensional space. Red: bacteria grown *in vitro* under acidic conditions (pH = 4); blue: bacteria grown *in vitro* under normal pH conditions (pH = 7.); green: short-term *in vivo* infection; black: long-term *in vivo* infection.


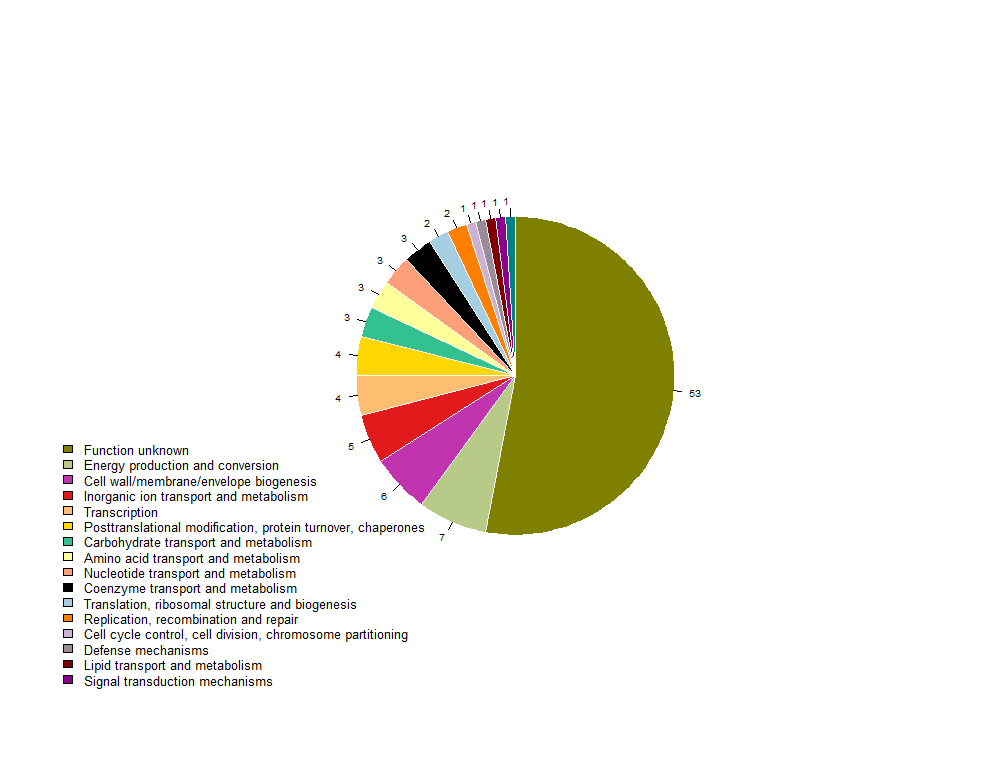


Figure S2: COG categories of genes (n = 588) in *B. melitensis* 16M that are specifically up- or downregulated in response to acidic stress.. COG categories were retrieved from the EggNOG database [36].
